# Modern Cookery, &c.

**Published:** 1845-04

**Authors:** 


### MODERN COOKERY IN ALL ITS BRANCHES REDUCED TO A SYSTEM OF EASY PRACTICE, &c. By *Eliza Acton*. Longman and Co. 1845.

AMONG the revolutions and improvements that have taken place in medicine during the present century, the increased attention to DIETETICS is one of the most important and conspicuous. We seldom take physic except when we are ill; but we are always taking food, and many of our maladies are induced by improper diet. We strongly suspect that our augmented longevity is as much owing to food as to physic. Cookery is, therefore, not beneath the notice of the physician. A late celebrated—at least fashionable—physician owed much of his popularity to his readiness in prescribing a dish as well as a draught for his patient:—and a living physician, of high literary and scientific attainments, is not less indebted to his chemistry of the kitchen, than to his pharmaceutical knowledge, for the high station he now holds. A knowledge of the cuisine is more necessary to the medical man than to any other professional man. His constant intercourse with dyspeptics—hypochondriacs—invalids and convalescents, calls upon him daily and hourly for advice as to diet; and he finds no small difficulty in adapting the dish to the nature of the complaint and the habit of the individual. Many a convalescent would fall back and perish were not food furnished of a kind that will suit the languid appetite and the morbidly fastidious palate.

Although England has long taken the lead in arts, commerce, and manufactures, she has made slow advance in the noble science of gastronomy, and has generally called in the assistance of France and Italy in the mysterious chemical processes going on in the lower regions of John Bull's castle. There JOHNNY CRAPEAUD rules the roast, sending up, daily, to the master of the mansion a farrago of "dishes tortured from their native taste," sufficient to feed the whole brood of Proteian devils that prey upon the vitals of the family, and give unfailing occupation to the doctor, the apothecary, and the undertaker! Everybody knows that God sends food to mankind, but that tbe cooks come from a very different quarter. The young doctor, therefore, who has often a young wife, and still younger children, should know whether he has "death in the pot," as well as dumplings. Economy in cooking is equally important to the rising generation of our brethren, as economy in dress, house-rent, and equipage—perhaps more so. We cannot, therefore, too warmly commend to the notice of our junior brethren this compilation of Eliza Acton, which will prove as useful to young Mrs. and her cook in the kitchen, as Thomson's Dispensatory or Conspectus to the young doctor in the library.

